# Long‐term performance monitoring of a‐Si 1200 electronic portal imaging device for dosimetric applications

**DOI:** 10.1002/acm2.14551

**Published:** 2024-10-07

**Authors:** Ivan Kutuzov, Ryan Rivest, Eric VanUytven, Boyd McCurdy

**Affiliations:** ^1^ Department of Physics and Astronomy University of Manitoba Winnipeg Manitoba Canada; ^2^ Medical Physics Department CancerCare Manitoba Winnipeg Manitoba Canada; ^3^ Department of Radiology University of Manitoba Winnipeg Manitoba Canada

**Keywords:** EPID, EPID dosimetry, EPID response stability, linac output, linac QA, machine QA, Radiotherapy

## Abstract

**Purpose:**

Recently, dosimetri applications of the electronic portal imaging device (EPID) in radiotherapy have gained popularity. Confidence in the robust and reliable dosimetric performance of EPID detectors is essential for their clinical use. This study aimed to evaluate the dosimetric performance of the a‐Si 1200 EPID and assess the long‐term stability of its response.

**Methods:**

Weekly measurements were performed on two clinically used TrueBeam linear accelerators (linacs) equipped with a‐Si 1200 EPID detectors over a 2‐year period. They included dark and flood calibration fields, and EPID response to an open field corrected for the long‐term machine output drift measured with the secondary absolute dosimeters: an ion chamber and an ion chamber array. All measurements were performed using five photon beam energies and two imaging modes: continuous and dosimetry. The measurements were analyzed for constancy and the presence of long‐term trends. Comparisons were made between the two linacs for each beam energy. Pixel sensitivity matrices (PSM) were determined semi‐annually and analyzed for long‐term constancy for both treatment machines.

**Results:**

The long‐term variation of the dark and flood field signals, integrated across the EPID plane, over the entire observation period did not exceed 0.17% and 0.79%, respectively. The output‐corrected EPID response showed long‐term variation from 0.28% to 0.36%, depending on beam energy, while the short‐term variation was 0.04%–0.07% for EPID and 0.02%–0.06% for secondary dosimeters. The long‐term variation of secondary dosimeters was 0.2%–0.3%. PSMs were found to be stable to within 1% for 97.8% of pixels and 2% for 100% of pixels.

**Conclusion:**

Techniques to monitor and assess the long‐term performance of the a‐Si 1200 EPID as a dosimeter were developed and implemented using two TrueBeam linacs. The long‐term variation of the EPID response was within clinical tolerance indicated in AAPM TG‐142 report, and the detector was shown to be stable and reproducible for routine clinical dosimetry.

## 1 INTRODUCTION

Electronic portal imaging devices have been established as two‐dimensional dosimetry systems in radiotherapy. There have been reports in the literature using the EPID for quality assurance (QA).^1^, ^2^, ^3^, ^4^, ^5^, ^6^, ^7^, ^8^, ^9^, ^10^, ^11^, ^12^, ^13^, ^14^, ^15^, ^16^ Applications include using the EPID for linac QA^1^, ^2^, ^3^, ^4^ and for pre‐treatment patient specific QA.^5^, ^6^, ^7^, ^8^ Several research groups have implemented various in vivo dose reconstruction algorithms based on transmission EPID images acquired during radiotherapy treatment sessions.^8^, ^9^, ^10^, ^11^, ^12^, ^13^ Recently, the possibility of using EPID as a real‐time dosimeter (i.e., during treatment) for delivered patient dose verification was described.^14^, ^15^, ^16^ Several commercially available EPID‐based solutions for clinical QA were listed in the AAPM TG‐307 report.^16^

EPID flat‐panel detectors have high spatial and temporal resolution, good signal reproducibility, and a linear dose response.^17^, ^18^ Aside from these parameters, institutions employing EPIDs for routine QA procedures or in vivo dosimetry must have confidence in the long‐term stability and reproducibility of the EPID response. Several research groups have published their findings on the stability of EPID dose response and other dosimetry‐related parameters.^19^, ^20^, ^21^, ^22^, ^23^, ^24^, ^25^, ^26^, ^27^

Barnes and Greer conducted daily monitoring of the machine performance check (MPC) procedure using the a‐Si 1200 EPID detector and compared the measured signal against reference dosimeters, over a 5‐month period.^25^ They investigated four available beam energies in a clinically used TrueBeam linac. They found that the MPC response showed a drift of 0.6% over the 5‐month observation period, while the independent machine output measurements using EPID were well correlated with the reference absolute dosimeter measurements.^25^

Pearson et al. compared the MPC output measurements using the a‐Si 1200 EPID detector for two photon beam energies with the monthly ionization chamber measurements over a 3‐year period on eight TrueBeam linacs.^26^ The authors found that the MPC output values agreed with the monthly ionization chamber comparison measurements for all beam energies within 1.5% in 97.8% of all measurements they made.^26^

Both abovementioned research groups used Varian TrueBeam machines and a‐Si 1200 EPID detectors, which are the same type of equipment used in this study. Their results correspond to the AAPM task group report TG‐142 that requires monthly x‐ray output measurements to be within 2% from the baseline.^28^ Both groups, however, focused on investigating the performance of MPC measurements for validating machine output constancy. MPC is an image‐based tool used for checking geometric and beam parameters of the TrueBeam linacs.^25^ While useful for verification of certain radiation beam parameters, this imaging mode is not necessarily useful for clinical dosimetry. The two EPID imaging modes commonly used for dosimetric purposes are continuous and dosimetry modes.^9^, ^10^, ^11^ They will be introduced later in the text. This study focuses on the investigation of the stability of EPID response when operated in those imaging modes.

Renaud and Muir compared variation of the long‐term stability of EPID response at the central pixel region reported by several groups.^27^ Several EPID models from three different manufacturers were described, the observation timelines were from five months to three years, the effect of linac output variation on EPID response was accounted for, and the relative variation was expressed as one standard deviation. The authors found standard deviation of EPID response to be within 0.2%–0.7%, which again corresponds to TG‐142 guidelines.

The purpose of this study was to consolidate and further extend the previous efforts made by other groups and conduct a comprehensive stability analysis of EPID dosimetric parameters over an extended period of time. To do this, weekly measurements of the EPID response were collected and compared against measurements made with absolute reference dosimeters, dark and flood fields were measured and analyzed on a weekly basis, and PSMs were determined and analyzed semi‐annually. All collected measurements were analyzed for long‐term constancy and to identify any possible trends.

To ensure the thoroughness and completeness of this study, the authors made sure that several conditions were simultaneously fulfilled. The experiment was conducted over the long‐term period of 24 months. Two clinically used treatment machines were involved to enable the intercomparison of the results. All available beam energies were explored. Dark and flood calibration fields were explored, as their measurements may influence the dosimetric outcomes when EPID is used as a dosimetry tool. Continuous and dosimetry image acquisition modes, important for EPID dosimetry applications, were explored separately. Analysis of the individual pixel measurements was made which included calculations of the pixel sensitivity matrices.

## 2 METHODS

The measurements were performed on two clinically used TrueBeam linacs (Varian Medical Systems, Palo Alto, California, USA) equipped with model a‐Si 1200s EPIDs. The local names of the linacs were “Unit A” and “Unit J,” and they will be further referred to using these names to distinguish between them. Weekly measurements were carried out over the 2‐year period between September 2019 and September 2021.

The a‐Si 1200 EPID model has a 43 × 43 cm^2^ active detector area consisting of 1280 × 1280 pixels. Two image acquisition modes, important for dosimetric EPID applications, were used when gathering weekly images. The first is continuous (or “cine”) mode, associated with time‐resolved imaging required when rotating the gantry during dose delivery. This mode is suited for RapidArc or volumetric‐modulated arc therapy (VMAT) dosimetry.^9^, ^10^, ^11^ The terms “continuous” and “cine” will be used interchangeably further in the text. When EPID is operated in continuous mode, all 1280 × 1280 pixels are used. The second imaging mode is dosimetry (or integrated) mode, which is suited for static‐gantry intensity‐modulated radiotherapy (IMRT) delivery.^10^ The terms “dosimetry” and “integrated” will also be used interchangeably further in the text. In dosimetry mode, only 1190 × 1190 pixels are used, which corresponds to an approximately 40 × 40 cm^2^ measurement area.

### 2.1 Dark field and flood field long‐term variation

The dark field and flood field calibration images were measured to assess their long‐term reproducibility. Measurements acquired on the first day of observation were used as baselines. The dark field image represents EPID signal measured in the absence of beam, while the flood field image represents EPID response to an open square field, large enough to cover the entire sensitive area of the detector. All five available beam energies were tested to assess calibration field reproducibility. This includes flattened 6, 10, and 23 MV beams (further denoted as 6X, 10X, 23X) and “flattening filter free” 6 and 10 MV beams (denoted as 6FFF, 10FFF). All possible combinations of field types, imaging modes, and energies were examined in each linac. When measuring flood fields, maximum dose rates were used, which were 600 MU/min for all flattened beams, 1400 MU/min for 6FFF beams, and 2400 MU/min for 10FFF beams.

Each collected dataset was processed as follows. First, measured images were integrated spatially across the imaging plane, and standard deviation of the integrated signal was calculated over the entire period of observation, to assess long‐term signal variation. The image integrals were plotted against time, and Pearson correlation coefficients were calculated pairwise between all energies to assess the level of correlation. Furthermore, a least‐squares linear fit was applied to each spatially integrated dataset, and the coefficients of determination were calculated between the measured and fitted data, to identify possible trends. Second, standard deviation over the entire observation period was calculated for each individual pixel. This quantity will be further referred to as a “pixelwise” standard deviation. The distributions of pixelwise standard deviations dataset were compared between beam energies and between two treatment machines.

### 2.2 Short‐term EPID response stability

Short‐term stability was evaluated for each detector used in this study, which included EPID operated in both imaging modes, and two reference detectors: a secondary ion chamber (Exradin A12, Standard Imaging) calibrated for absolute dose measurement and an ion chamber array (MatriXX; IBA Dosimetry, Swarzenbruck, Germany). All measurements were performed on the same day.

Each reference detector was irradiated with an open square field, using all five available energies, five times per energy, thus making a series of 25 irradiations per detector. A 100 MU per measurement was delivered, and maximum dose rates, mentioned in the previous section, were used for each beam energy. Absolute ion chamber measurements were made using the TG‐51 setup, which includes 10 × 10 cm^2^ open field and source‐to‐axis distance (SAD) of 100 cm with 10 cm solid water buildup and 10 cm backscatter layers. MatriXX measurements were made using a manufacturer recommended QA profile measurement setup, which includes 76.2 cm source‐detector distance (SDD) and field size of 30 × 30 cm^2^ at isocenter, with a 5 cm, water‐equivalent buildup layer.

The EPID measurement series was repeated twice: once for continuous mode and once for dosimetry mode. The continuous mode measurements used the standard continuous flood field measurement setup: 30 × 30 cm^2^ field at isocenter and SDD of 150 cm, which is equivalent to 45 × 45 cm^2^ field size at the EPID plane and covers the entire sensitive area of the detector. The dosimetry mode measurements used the standard dosimetry flood field measurement setup: 40 × 40 cm^2^ setup with a SDD of 100 cm, which also cover the entire sensitive area of the detector operated in dosimetry mode. Five measurements using 100 MU were made for each energy in each imaging mode, using maximum dose rate, which resulted in a total of 50 EPID measurements.

Standard deviation of the measured signal calculated over the series of the same day measurements was used to characterize reproducibility of each detector. Standard deviations were calculated for the beam central axis (CAX) area for all detectors, and for the entire detector area for EPID and MatriXX detectors. For EPID, standard deviation was calculated for the signal integrated over the central 1 cm^2^ area, which corresponds to adding signals from 900 central pixels (30 × 30‐pixel region) in the EPID detector. For MatriXX, signal summarized over the four central ion chamber was used for calculations. The results were compared with corresponding standard deviations observed for the ion chamber measurements. Also, standard deviations of signal averaged over the entire plane were compared for MatriXX and EPID used in both imaging modes.

### 2.3 Long‐term EPID response stability

Weekly measurements of the EPID response against a secondary ion chamber dosimeter positioned on the central axis, and an ion chamber array centered on the central axis, were carried out to evaluate the EPID response variability over the entire observation period. The output values measured with EPID were corrected for the natural linac output fluctuations, assessed with the reference dosimeters on the same day. The corrected EPID output measurements were analyzed using standard deviation calculated over the entire observation period.

Linac output measurements using a single ion chamber were performed at a 100 cm SAD setup, with a 10 cm solid water buildup and a 10 cm solid water backscatter layer. The ion chamber was positioned at isocenter, field size of 10 × 10 cm^2^ at isocenter was used, and 100 MU were delivered using the maximum dose rate for each beam energy. The dose was measured three times for each energy, and the average value read by electrometer was used as the measured linac output. Then, the EPID was positioned at a 100 cm SSD, and a 10 × 10 cm^2^ field at isocenter was acquired using 100 MU and the maximum dose rate for each beam energy, using the dosimetry acquisition mode. The EPID image pixel measurements averaged over a 1 × 1 cm^2^ central area around the beam central axis were used as the EPID response to the same reference beam.

Linac output measurements using a 2D ion chamber array were performed at a 76.2 cm SDD setup, following the manufacturer guidelines for beam profile measurements. The MatriXX detector was positioned in the manufacturer supplied gantry holder, and a field size of 30 × 30 cm^2^ at isocenter was used, which covers the entire ion chamber array (24.4 × 24.4 cm^2^) at that SDD. Again, 100 MU was delivered at the maximum dose rate, and a two‐dimensional beam profile was measured for each beam energy. After removing the MatriXX and holder, and using the same field size and dose rate, an EPID image was acquired using dosimetry mode at a larger SDD of 130 cm, which corresponds to a projected field size of 39 × 39 cm^2^ at the EPID plane at this SDD. The EPID signal integrated over the entire detector was used as the EPID response value, and the MatriXX signal integrated over the area of the entire array was used as the response of the ion chamber array to the same reference beam.

The linac output measurements made in the first week with the reference absolute dosimeters were taken as a baseline. The EPID measured output values were corrected for the machine output fluctuations using the reference dosimeter measurements. The corrected EPID output measurements were analyzed using standard deviation over the entire 2‐year observation period.

### 2.4 Pixel sensitivity matrix variation

Pixel sensitivity matrices indicate individual pixel gain variations across the EPID and are an important correction distribution needed for many dosimetric applications of the EPID. PSMs were measured semi‐annually using the method described by Greer and Barnes.^29^, ^30^ Using this method, individual gains of each pixel are expressed as a relative value in terms of one reference pixel (usually located in the center of EPID array), whose gain is set to unity. PSMs obtained for each linac over the observation period were compared to assess relative changes in the calculated individual pixel sensitivity using percentage difference.

## 3 RESULTS

### 3.1 Dark field long‐term variation

#### 3.1.1 Dosimetry imaging mode

**FIGURE 1:**
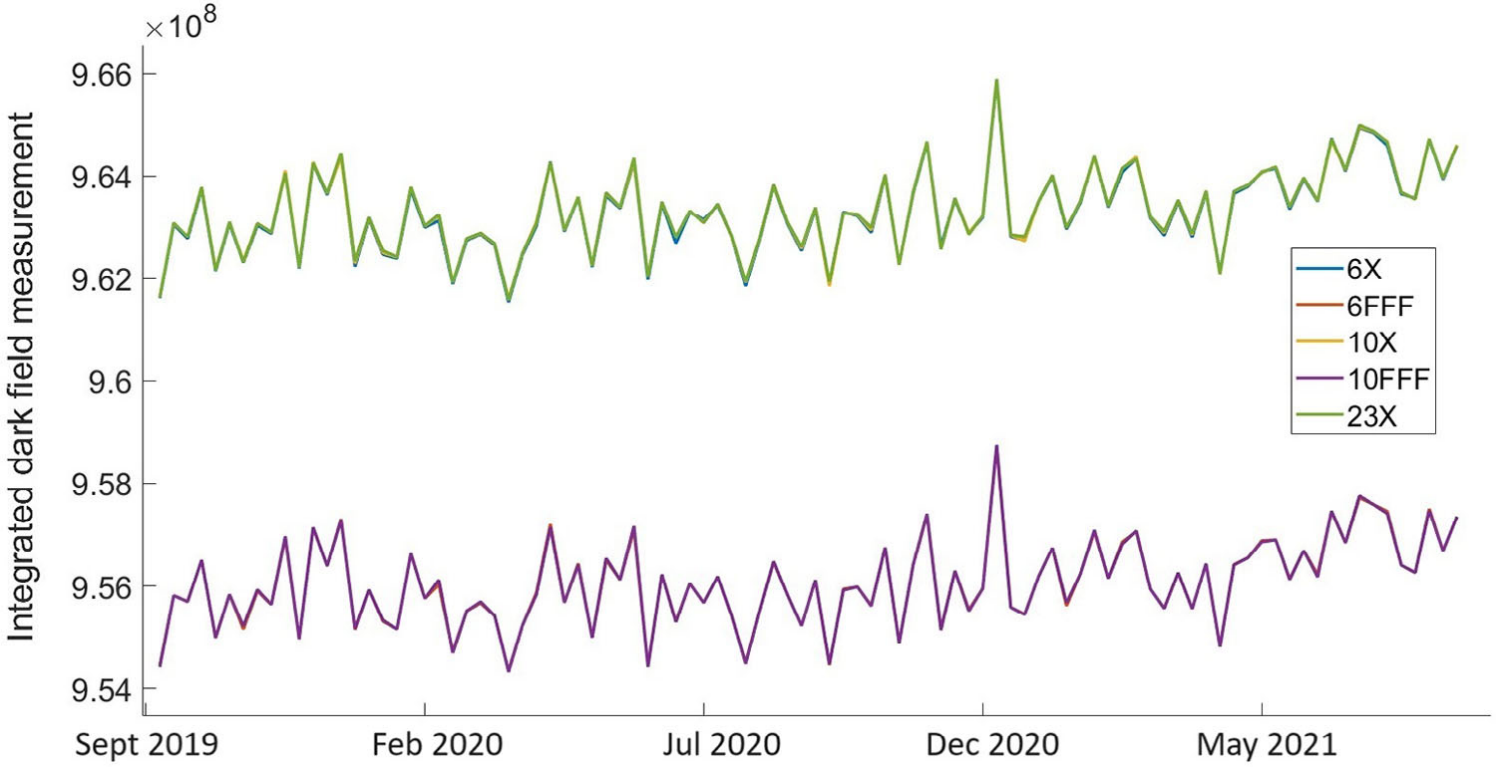
Integral dark field measurements for all beam energies, dosimetry mode, Unit A. Note that data for the FFF beams overlay each other on the lower line while data for the non‐FFF beams overlay each other on the higher line.

Figure 1 shows the dosimetry mode dark field signal integrated over the entire imaging plane, as observed on Unit A over the entire two‐year period, for all beam energies. As one can see from Figure 1, all five beam energies are very well correlated with one another. In fact, the plots for three beam energies that use a flattening filter (6X, 10X, and 23X) are almost coincident, as are the plots for both FFF beam energies (6FFF and 10FFF). The dark fields measured for Unit J demonstrate similar behavior (data for Unit J is not shown in Figure 1).

The values of Pearson correlation coefficients calculated pairwise between all five beam energies were found to be within the interval 0.994≤r≤0.999 for Unit A and in the range of 0.997≤r≤0.999 for Unit J. The values found for both machines are very close to the ideal value of r=1. The dosimetry mode dark fields integrated over the detector plane showed very small fluctuations over the 2‐year observation period. The calculated standard deviations from plots in Figure 1 were in the range between 0.10% and 0.11%, depending on the beam energy. Integrated dark field measurements on Unit J showed similar results with the standard deviation being in the range between 0.16% and 0.17%.

Dark field measurements fitted using the least squares linear fit did not demonstrate a clear trend. For both machines and all energies, the fitted lines showed an almost negligible increase of about 0.10%–0.14% over the 2 years of observation, and a small coefficient of determination, $R^{2}$. The coefficients of determination ranged $0.15\leq R^{2}\leq 0.17$ for flattened beams and were in the range of $0.11\leq R^{2}\leq 0.13$ for FFF beams. This speaks in favor of random fluctuations in the dark field measurements rather than a trend despite a very small increase observed over 2 years.

Across all beam energies, Unit A had a range of calculated pixelwise standard deviations from 0.08% to 0.32% with the average of 0.16%, while Unit J ranged from 0.10% to 0.35% with the average of 0.21%. The observed dark signal variation can differ noticeably depending on the location of the pixel. The calculated pixelwise standard deviations have greater differences in the crossplane direction than they do in the inplane direction.

**FIGURE 2:**
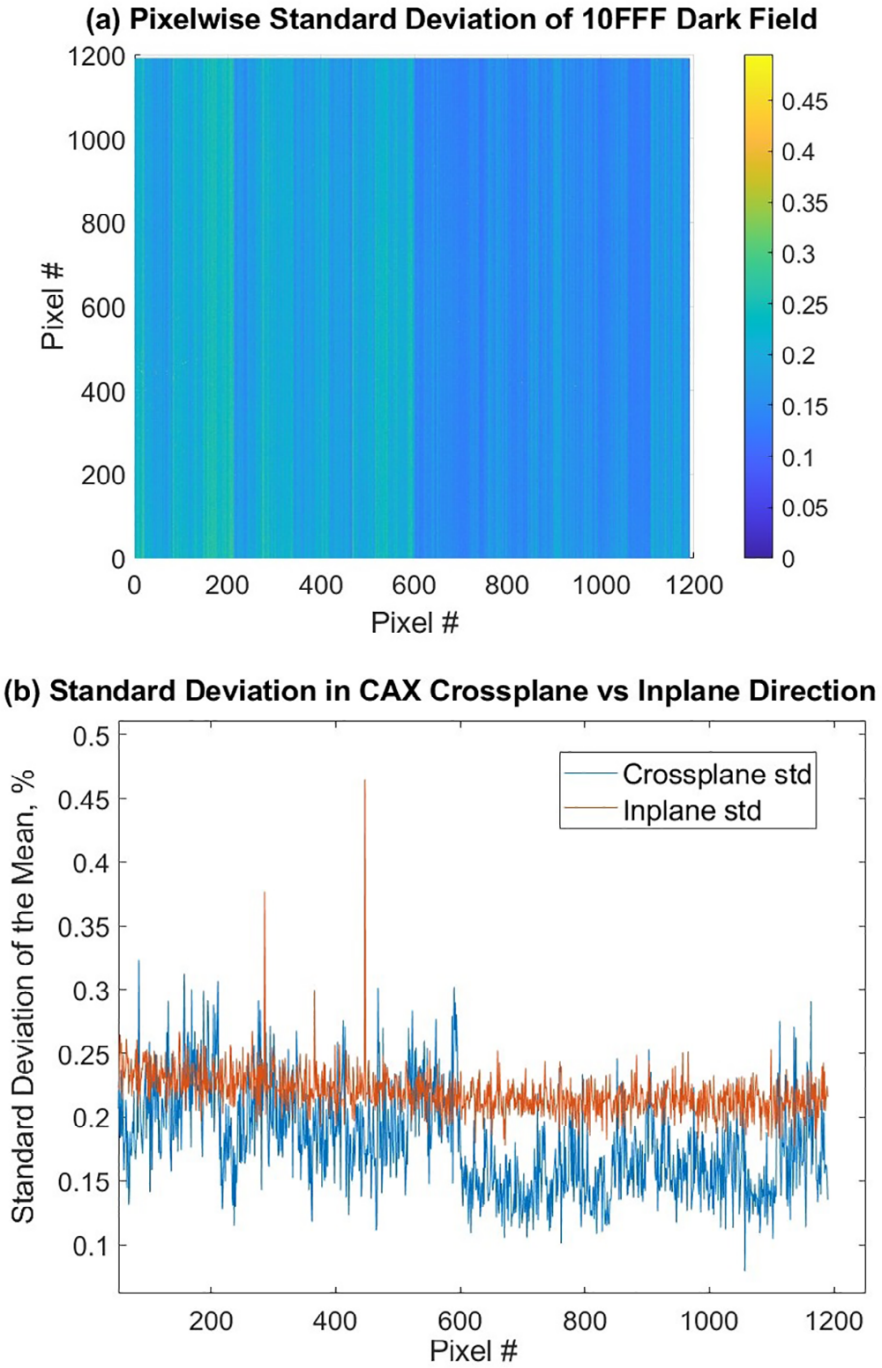
(a) Pixelwise standard deviation for dosimetry 10FFF dark field on Unit A, (b) CAX crossplane and inplane profiles of data in (a).

Figure 2 demonstrates the difference in signal variation along these directions for the 10FFF dosimetry dark field measured on Unit A. Figure 2a shows a colorplot of the dark field standard deviation across the entire EPID plane. Figure 2b demonstrates the central axis values plotted in both directions. Two‐dimensional patterns similar to Figure 2a were observed in both machines for all beam energies.

#### 3.1.2 Continuous imaging mode

The continuous mode dark fields integrated over the imager plane and observed over the entire observation period were found to be well correlated between all tested beam energies, in both machines. The pairwise Pearson correlation coefficients for all pairs of measurements were in the range of 0.996≤r≤0.998 for Unit A and in the range of 0.994≤r≤0.996 for Unit J. Integral measurements for all five beam energies show a small standard deviation of the mean over the entire observation period, approximately 0.06% for Unit A, and 0.04% for Unit J.

The continuous mode dark field measurements integrated over the imager plane did not demonstrate any significant trend over time. The data fitted using the least squares linear fit only showed a negligible increase of about 0.01%–0.02% over 2 years, for all beam energies, in both linacs. The coefficients of determination ranged in the interval $0.012\leq R^{2}\leq 0.020$ in both machines, depending on the beam energy. This indicates that random fluctuations dominate the change in measured dark field signal over time.

Across all beam energies, Unit A had a range of calculated pixelwise standard deviations between 0.08% and 0. 21% with the average of 0.13%, while Unit J ranged from 0.07% to 0.20% with the average of 0.12%. The distributions of pixelwise continuous dark field standard deviations showed similar shapes for all energies, in both linacs.

It was found that the dark field measurements integrated over the imager plane and observed over time are correlated between the imaging modes, for all energies. Table 1 shows the values of Pearson correlation coefficient between the two modes for both machines and all beam energies.

**TABLE 1:** Correlation coefficient r between dosimetry and continuous dark field measurements.

Unit/Beam energy	6X	6FFF	10X	10FFF	23X
Unit A	0.85	0.88	0.85	0.88	0.86
Unit J	0.87	0.89	0.84	0.86	0.81

### 3.2 Flood field long‐term variation

#### 3.2.1 Dosimetry imaging mode

**FIGURE 3:**
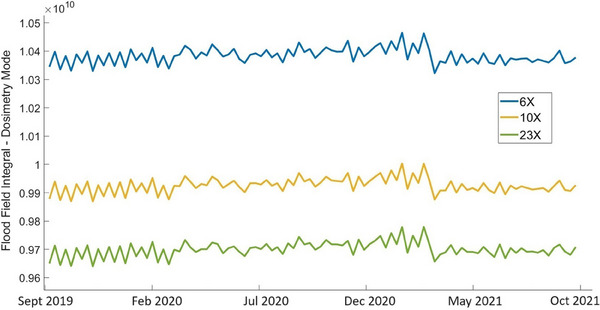
Dosimetry flood field measurements for flattened beams 6X, 10X, and 23X, Unit A.

Figure 3 shows the variation of the dosimetry flood field integrated over the imager area, as a function of time. The plots demonstrate measurements acquired over the entire observation period for all flattened beam energies. The FFF beams show very similar trends, but their magnitudes are greater than those of flattened beams by a factor of 2.2–2.3. Hence, data related to FFF beams is not shown in Figure 3. Calculations based on the dosimetry flood field measurements show that all five beam energies are very well correlated with one another. The values of Pearson correlation coefficient between flood field measurements for different energies calculated pairwise are in the range 0.94≤r≤0.99 for Unit A, and in the range 0.92≤r≤0.99 for Unit J.

Dosimetry flood field measurements shown in Figure 3 demonstrate greater fluctuations over the 2‐year observation period than the dark fields measurements (Figure 1). The standard deviations of the mean calculated for all beam energies are in the range from 0.43% to 0.46% for Unit A, and from 0.45% to 0.48% for Unit J. The best linear fits of the dosimetry flood field integrals, obtained using the least square method, show a small increase over 2 years, from 0.2% to 0.5% for Unit A and from 0.1% to 0.5% for Unit J, depending on the beam energy. Also, the best linear fits show small values of the coefficient of determination for all beam energies, with $0.05\leq R^{2}\leq 0.14$. for Unit A and $0.02\leq R^{2}\leq 0.11$ for Unit J. This indicates that random fluctuations dominate the flood field measurements, that is, there is no clearly determinable trend.

**FIGURE 4:**
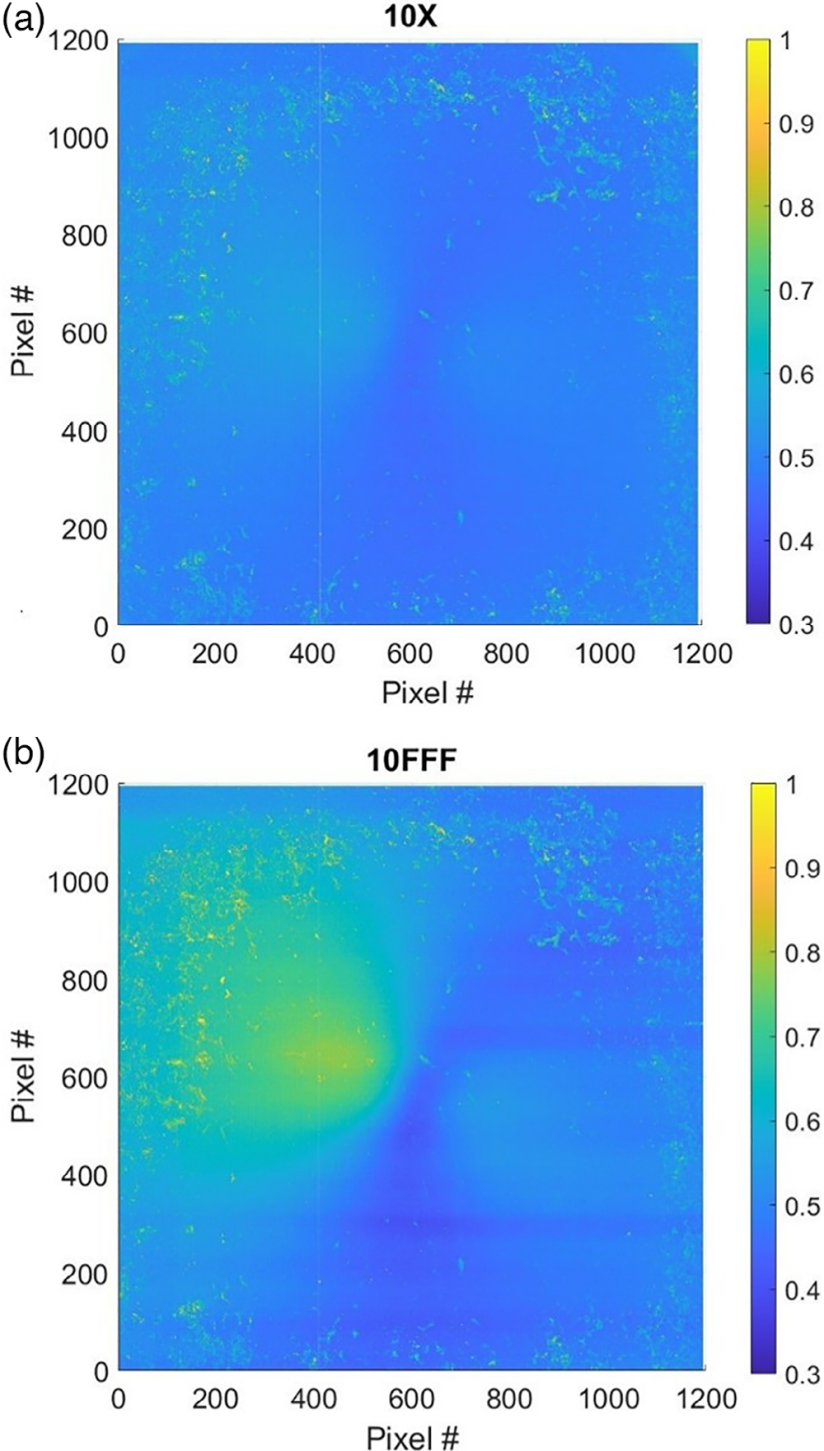
Color plot of pixelwise dosimetry flood field standard deviations, Unit J: (a) 10X beam and (b) 10FFF beam.

Figure 4 shows color plot of the distributions of pixelwise standard deviations for 10X and 10FFF beam energy on Unit J, on the same scale. One can notice that, although the patterns look similar, the 10FFF plot has a brighter area on the left part of the image. Also, for both beams, some pixels at the edges of the detector show greater standard deviation than those in the central region. Other than that, the EPID detectors do not demonstrate any areas with significantly higher or lower than average values of dosimetry flood field standard deviation.

**FIGURE 5:**
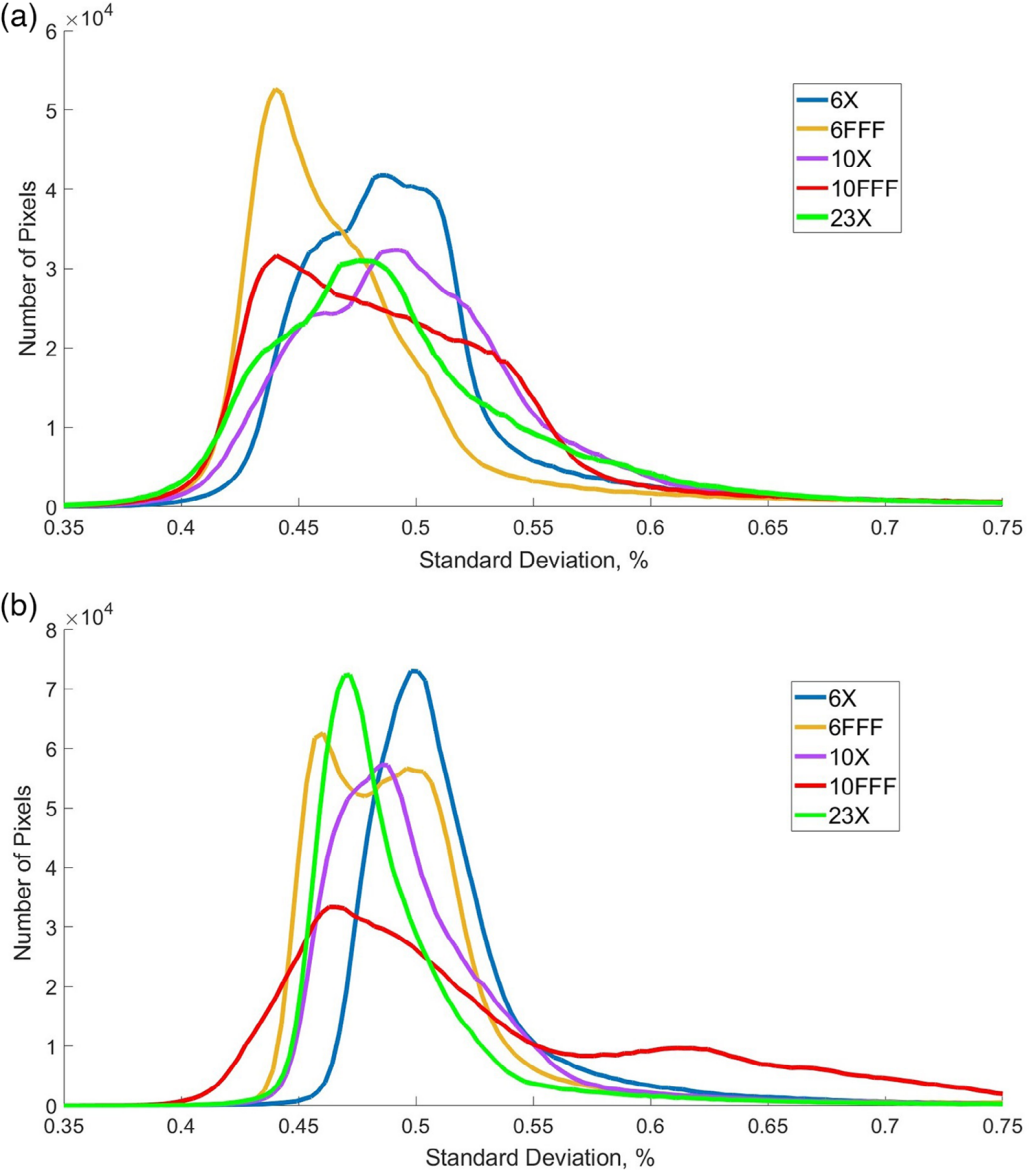
Distributions of standard deviations of individual EPID pixel flood field measurements in dosimetry mode for all beam energies, for Unit A (a) and Unit J (b).

Figure 5 demonstrates distributions of pixelwise standard deviations of the dosimetry mode flood field measurements calculated across the EPID plane for both linacs. Most pixels in both machines have standard deviations between 0.35% and 0.70%. The only exception is 10FFF beam on Unit J, which has a second mode between 0.60% and 0.65% with a “tail” that extends beyond 0.70%. This part of the distribution may correspond to the area with higher values of standard deviation shown for that energy in Figure 4b. All other beam energies for Unit J (Figure 5b), as well as all five beam energies on Unit A (Figure 5a), have color plot of their standard deviations similar to the one shown in Figure 4a, for 10X beam on Unit J.

Table 2 shows magnitudes of pixelwise standard deviations of measured flood field signal for all beam energies, averaged across the entire EPID plane. One can see from Table 2 that Unit J systematically demonstrates greater values of standard deviation than Unit A in dosimetry mode. However, the values for both machines and all energies have the same order of magnitude.

**TABLE 2:** Pixelwise standard deviation of measured flood field signal averaged across the EPID.

Imaging mode and linac name	Beam energy	6X	6FFF	10X	10FFF	23X
Dosimetry, Unit A		0.55%	0.53%	0.56%	0.55%	0.54%
Dosimetry, Unit J		0.63%	0.62%	0.61%	0.66%	0.59%
Continuous, Unit A		0.81%	0.71%	0.56%	0.40%	0.73%
Continuous, Unit J		0.65%	0.58%	0.64%	0.58%	0.56%

#### 3.2.2 Continuous imaging mode

**FIGURE 6:**
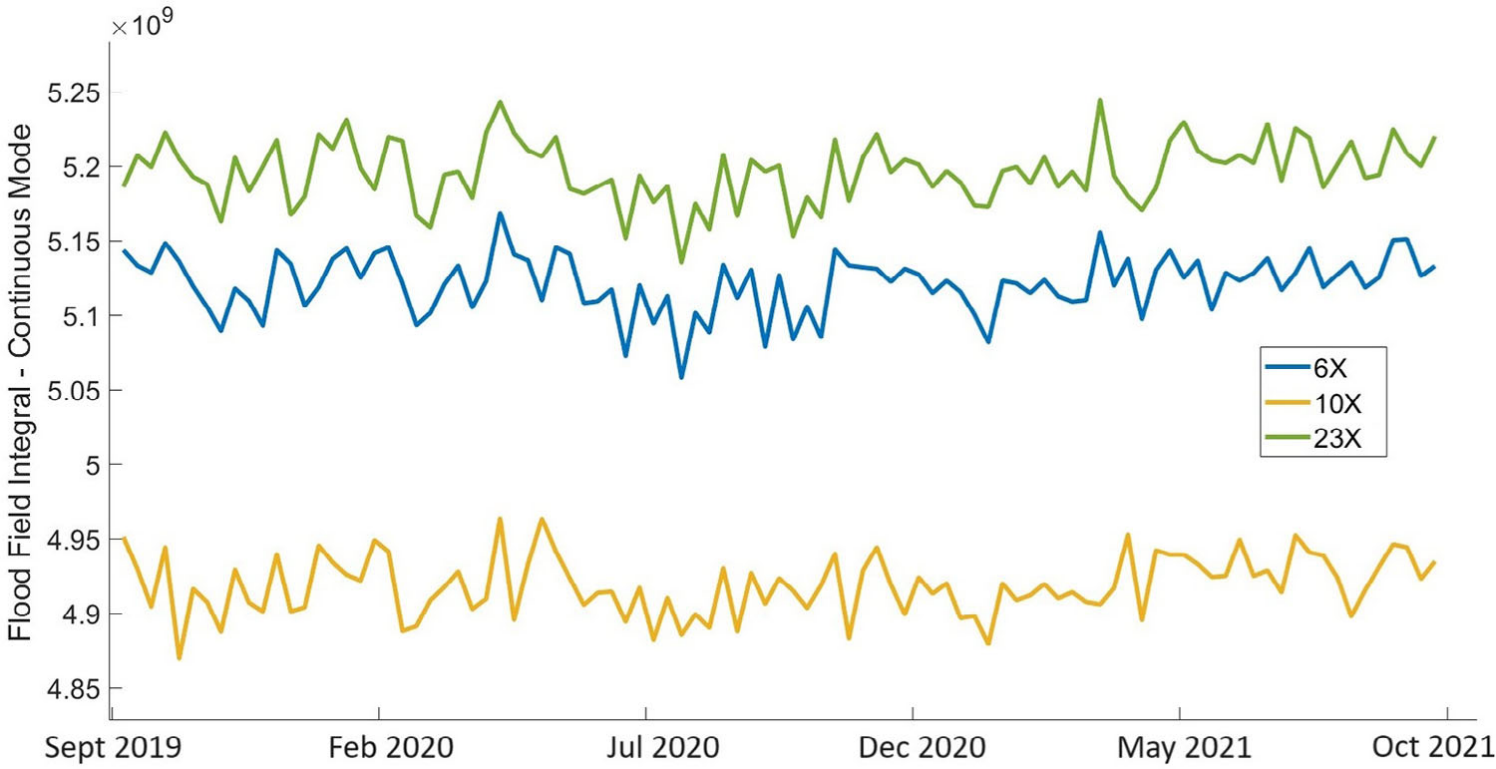
Continuous flood field measurements for flattened beams 6X, 10X, and 23X, Unit J.

Continuous flood field measurements integrated over the entire EPID plane and traced over the entire observation period are less corelated between the beam energies (in both machines) than the dosimetry flood field measurements. The values of Pearson correlation coefficient between flood field measurements for different energies calculated pairwise are in the range 0.57≤r≤0.71 for Unit A, and in the range 0.66≤r≤0.72 for Unit J. Figure 6 shows plots of the continuous flood field measurements integrated over the entire imaging plane on Unit J. Only the measurements related to the flattened beams (6X, 10X, and 23X) are shown since the FFF beams have magnitudes that differ significantly from the flattened beams, and, hence, their plots do not fit the scale.

The continuous flood field measurements shown in Figure 6 demonstrate fluctuations over the 2‐year observation period. The standard deviations of the mean calculated for all beam energies are in the range from 0.49% to 0.79% for Unit A, and from 0.53% to 0.62% for Unit J.

The flood field readings demonstrate a small drift over 2 years, less than 0.1% for both machines and all beam energies. The best linear fits of the continuous flood field integrals show small values of the coefficient of determination for all beam energies, with $0.04\leq R^{2}\leq 0.07$. for Unit A and $0.03\leq R^{2}\leq 0.06$ for Unit J. This indicates that random fluctuations dominate variations of the flood field measurements.

**FIGURE 7:**
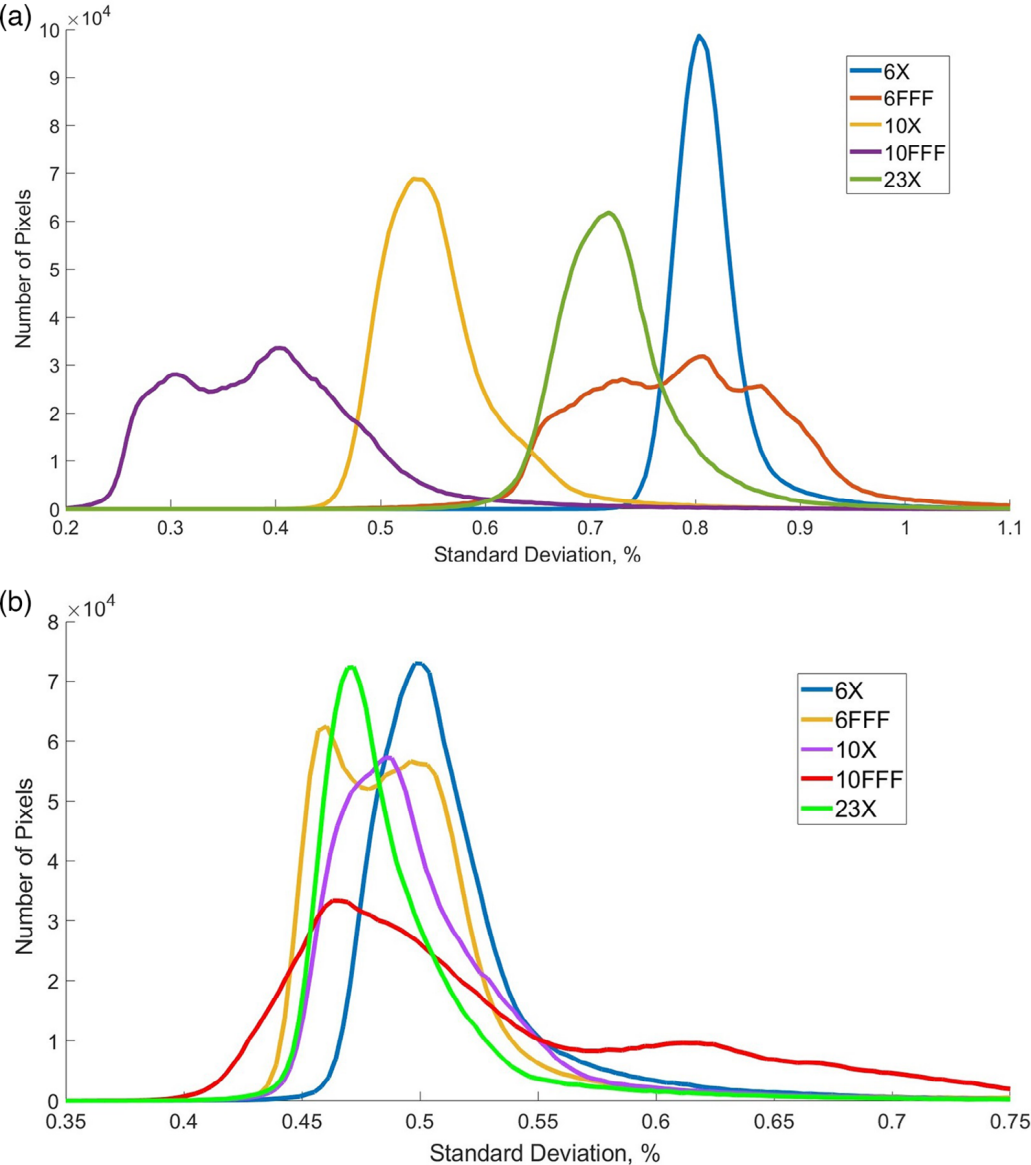
Distributions of standard deviations of individual EPID pixel flood field measurements in continuous mode for all beam energies, for Unit A (a) and Unit J (b).

Figure 7 demonstrates distributions of pixelwise standard deviations of the continuous mode flood field measurements calculated across the EPID plane for both linacs. The values of pixelwise standard deviations averaged across the EPID plane, for all investigated beam energies, are shown in Table 2. One can see that the magnitudes of standard deviations have similar values for both imaging modes. However, as follows from the comparison of Figures 4 and 7, the shapes of their distributions are different. All flattened beams in continuous mode demonstrate monomodal, gaussian‐like distributions of pixelwise standard deviation, while all‐non‐flattened beams show bi‐modal, or multimodal distributions. If plotted in 2D plane, all flattened beams in both machines show color plots of pixelwise standard deviation similar to the one in Figure 4a, while the non‐flattened beams show color plots similar to the one in Figure 4b.

### 3.3 Short‐term EPID response stability

Table 3 shows the comparison of the short‐term CAX standard deviations calculated for ion chamber and central areas of MatriXX and EPID, for both imaging modes. Values in Table 3 are presented as a percentage of the mean signal value. These standard deviations have the same order of magnitude for the ion chamber, MatriXX, and EPID operating in dosimetry mode. However, EPID operated in continuous mode demonstrates values about one order of magnitude higher.

**TABLE 3:** CAX standard deviation calculated for ion chamber and central detector areas.

Detector/Energy	6X	6FFF	10X	10FFF	23X
Ion chamber	0.03%	0.05%	0.03%	0.05%	0.03%
MatriXX	0.02%	0.06%	0.02%	0.04%	0.02%
EPID dosimetry	0.04%	0.05%	0.06%	0.06%	0.04%
EPID continuous	0.52%	0.30%	0.55%	0.32%	0.37%

The standard deviations for all three detectors have comparable values from 0.02% to 0.06% if EPID is operated in dosimetry mode. The flattened beams show smaller standard deviations than the FFF beams when measured with ion chamber or ion chamber array. The linac output fluctuations (defined as the standard deviation) do not exceed 0.06%. Uncertainty of measurements using all three detectors cannot exceed 0.06%, either.

Table 4 shows comparison of standard deviation averaged over the entire detector area, for both MatriXX and EPID. Values in Table 4 are shown as percentage of the mean. One can notice that standard deviations of MatriXX and EPID operating in dosimetry mode have same order of magnitude; however, the EPID values are higher. On the other hand, the values demonstrated by EPID operating in continuous mode are greater by one order of magnitude.

**TABLE 4:** Standard deviation averaged over entire detector area for MatriXX and EPID.

Detector/Energy	6X	6FFF	10X	10FFF	23X
MatriXX	0.02%	0.03%	0.02%	0.04%	0.02%
EPID dosimetry	0.04%	0.06%	0.04%	0.07%	0.04%
EPID continuous	0.50%	0.32%	0.53%	0.33%	0.38%

As one can see from Table 4, flattened beams show smaller fluctuations compared to flattening‐filter free beams. Standard deviations measured by MatriXX equal 0.02% for flattened beams and are between 0.03% and 0.04% for FFF beams. As these values were measured using an absolute reference detector, the short‐term linac output fluctuations should not exceed them.

EPID measurements show standard deviation of 0.04% for flattened beams, and 0.06%–0.07% for FFF beams, which is approximately two times higher than for the MatriXX detector.

### 3.4 Long‐term EPID response stability

The output corrected EPID response measurements observed over the entire 2‐year period demonstrate correlation between different beam energies in both machines. The values of pairwise correlation coefficients for central EPID area measurements are in the range 0.971≤r≤0.986 for Unit A, and in the range 0.951≤r≤0.982 for Unit J. For the entire EPID measurements, they are in the range 0.953≤r≤0.977 for Unit A, and in the range 0.936≤r≤0.977 for Unit J.

Table 5 shows standard deviations of the corrected and uncorrected EPID response values observed in each machine for each beam energy. The uncorrected standard deviations are shown in parentheses, and the corrected standard deviations are shown without parentheses.

**TABLE 5:** Standard deviation of the measured EPID response (before) and after output correction, %.

Linac name, Ref. Detector	Beam energy	6X	6FFF	10X	10FFF	23X
Unit A, Ion chamber		0.34% (0.49%)	0.32% (0.48%)	0.29% (0.46%)	0.35% (0.49%)	0.28% (0.45%)
Unit A, MatriXX		0.31% (0.51%)	0.33% (0.49%)	0.29% (0.47%)	0.36% (0.50%)	0.30% (0.47%)
Unit J, Ion chamber		0.28% (0.45%)	0.34% (0.45%)	0.31% (0.44%)	0.34% (0.44%)	0.29% (0.44%)
Unit J, MatriXX		0.30% (0.46%)	0.35% (0.47%)	0.32% (0.45%)	0.36% (0.47%)	0.31% (0.46%)

**FIGURE 8:**
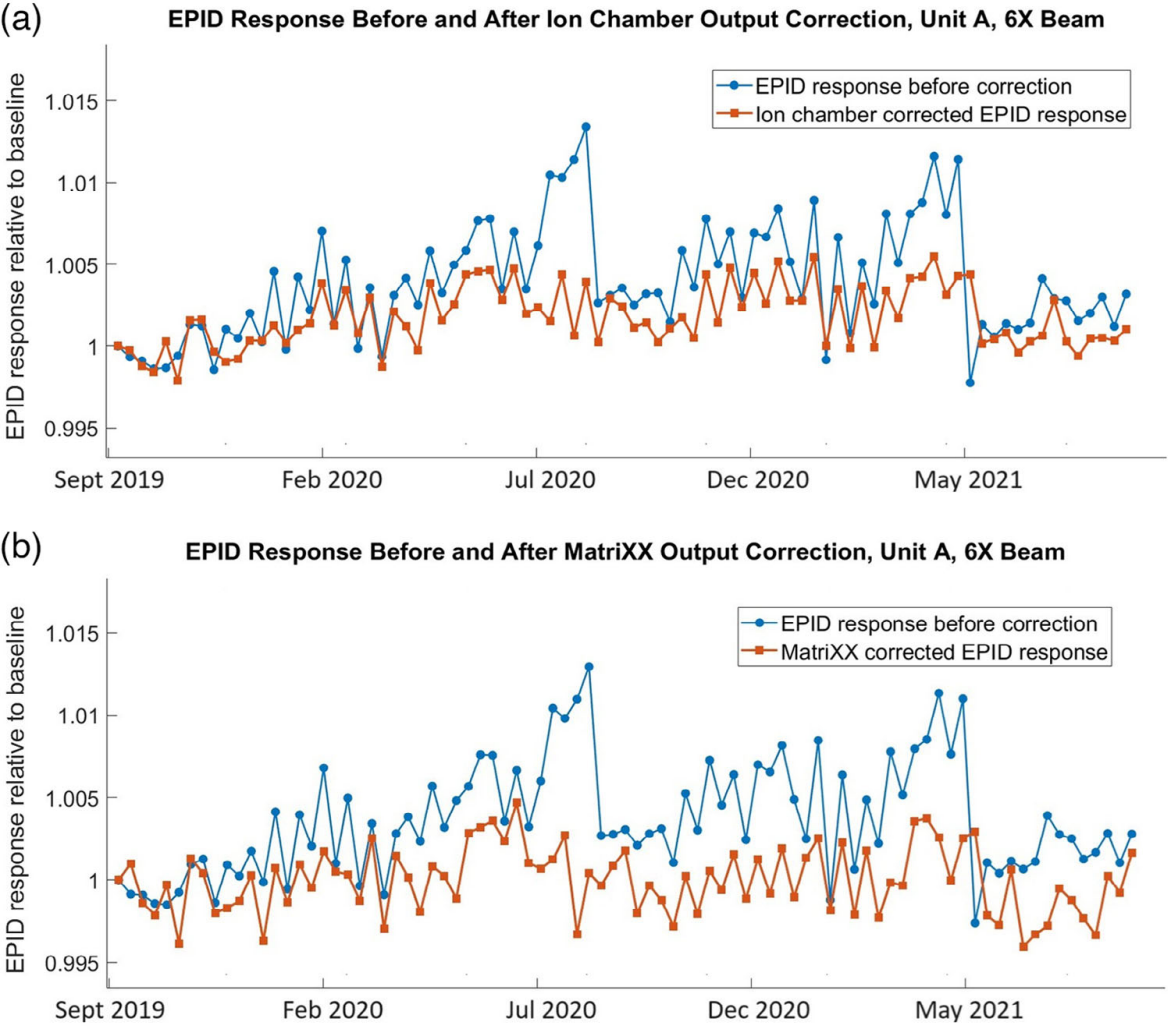
EPID measured 6X beam output, Unit A, uncorrected and corrected for the machine output using (a) ion chamber and (b) MatriXX measurements.

Figure 8 demonstrates variation of the EPID response (relative to the baseline) over the entire observation period. It also demonstrates EPID response correction for the machine output using ion chamber measurements (Figure 8a) and MatriXX measurements (Figure 8b). The figure uses EPID response to 6X beam on Unit A as an example. However, the other beam energies show similar results (Table 5). The standard deviation in this example was reduced from 0.49% to 0.34% by using ion chamber correction and from 0.51% to 0.31% by using MatriXX correction.

**FIGURE 9:**
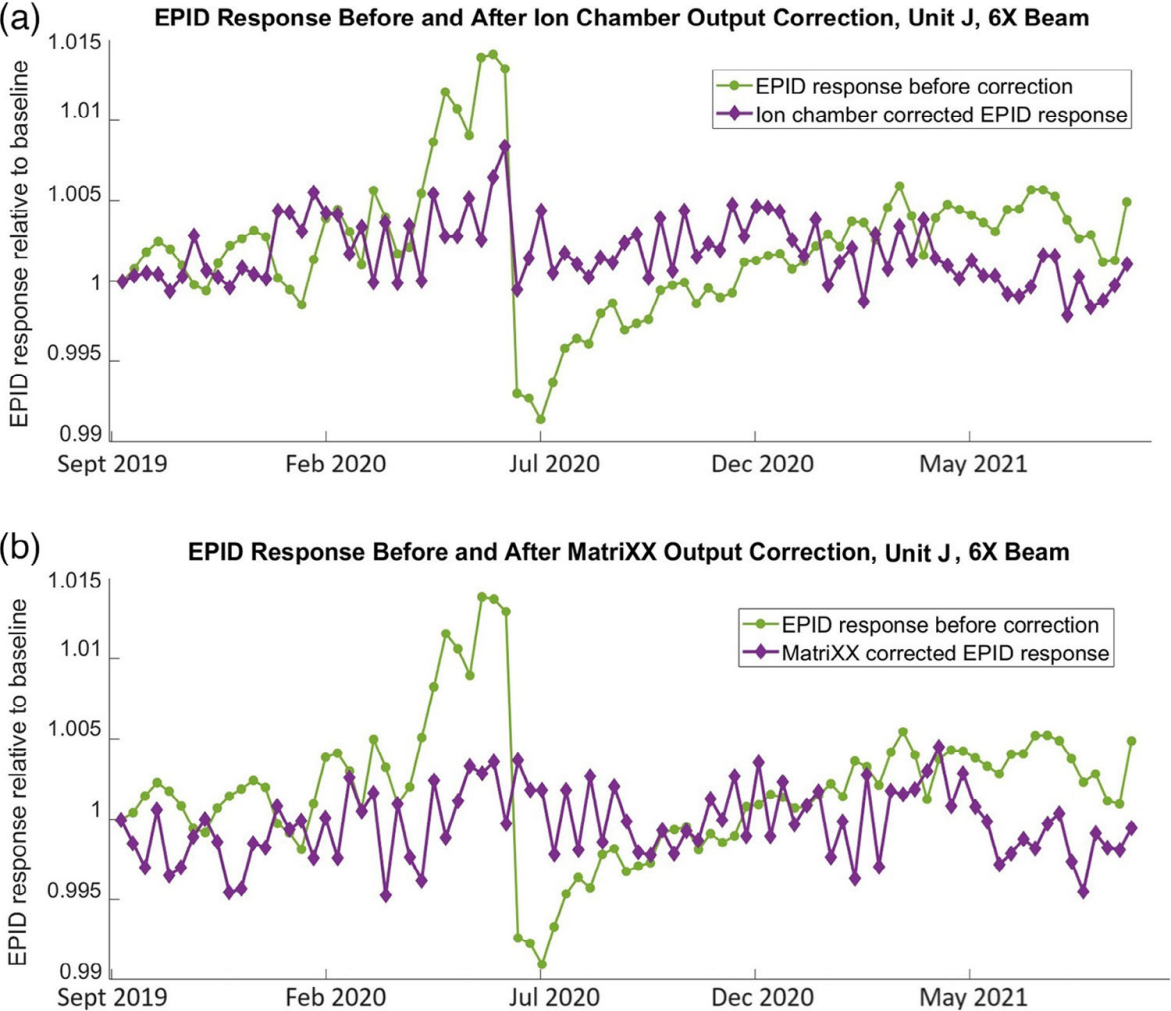
EPID measured 6X beam output, Unit J, uncorrected and corrected for the machine output using (a) ion chamber and (b) MatriXX measurements.

Figure 9 demonstrates variation of the EPID response (relative to the baseline) over the entire observation period, as well as the response correction for the machine output using ion chamber measurements (Figure 9a) and MatriXX measurements (Figure 9b). This example uses 6X beam on Unit J. The other beam energies on this machine show similar results (Table 5).

One can see from Figures 8 and 9 and Table 5 that, despite the machine output correction using absolute reference detectors, EPID detector still represents long‐term response variation with standard deviation in the range from 0.2% to 0.4%.

### 3.5 Pixel sensitivity matrix variation

**FIGURE 10:**
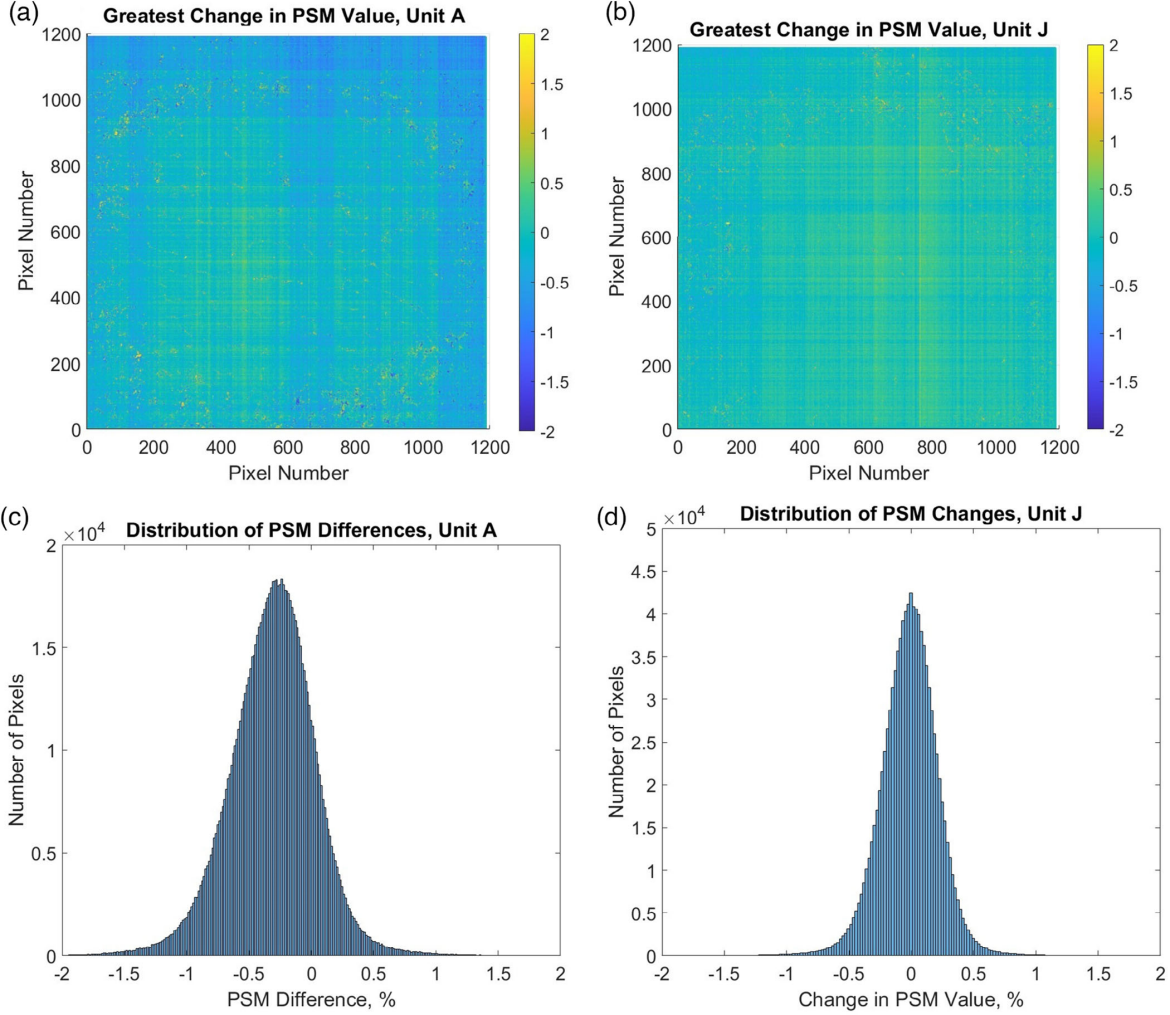
Colorplot of greatest pixelwise changes observed in PSM for (a) Unit A and (b) Unit J, and Distribution of greatest pixelwise changes observed in PSM for (c) Unit A and (d) Unit J.

Figure 10a demonstrates colorplots and distributions of greatest pixelwise changes in PSM observed over the entire 24‐month period, in both linacs. One can see from the figure that EPID detector does not demonstrate significant changes in its relative pixel sensitivity, as well as there are no detector areas that show significantly greater or lower change in PSM compared to the rest of detector area.

In Unit A, the magnitude of change in pixel sensitivity, averaged over all pixels, equals 0.37%, while in Unit J, this value is 0.18%. In Unit A, 97.8% of pixels showed changes below 1%, and 100% of pixels showed changes below 2%, over the period of observation. In Unit J, 99.3% of pixels have changed their sensitivity by 1% or less, while 100% of pixels changed by less than 2%.

## 4 DISCUSSION

Dark field measurements demonstrated high reproducibility over the 2‐year observation period. Dark field signals integrated over the entire EPID plane demonstrated small long‐term fluctuations, not exceeding 0.11% and 0.17% for Unit A and Unit J, respectively. These fluctuations may be partly due to the variable ambient room temperature and EPID operational temperature.^27^ It was found that, in both linacs and both imaging modes, the pixelwise standard deviations have greater variation from pixel to pixel in the crossplane direction than in the inplane direction. This difference can be attributed to the intrinsic properties of the EPID electronics.^19^

Dosimetry dark signal images also show exceptionally good correlation between beam energies, with the minimum correlation coefficient r=0.994 for both imaging modes, in both machines. At the same time, dark field measurements show very weak linear regression and do not demonstrate any clear trend. There is a small long‐term increase in dark field readings over the two‐year period, which can be a result of gradual radiation damage of the EPID detector.^19^ Correlation was found between dark fields measurements in dosimetry and continuous imaging modes in both machines, with the minimum correlation coefficient r=0.85 for Unit A and r=0.81 for Unit J.

Flood field measurements exhibit consistent reproducible behavior over the two‐year observation period. When integrated across the entire plane, flood field signals displayed long‐term fluctuations, with the standard deviation not exceeding 0.79% in Unit A and 0.62% in Unit J. The standard deviations of flood field signals exhibit higher values and a broader pixelwise spread compared to the dark field results. The variability of the flood field is influenced by both the stability of the electronic circuit and long‐term machine output fluctuations, leading to a greater standard deviation. In contrast, the reproducibility of the dark field is influenced solely by the long‐term stability of the EPID electronic circuit.

The pixelwise standard deviations of flood field signals exhibit a wider spread in continuous mode compared to dosimetry mode in both linacs. Notably, certain pixels at the detector's edges display higher standard deviations than those in the central region across both imaging modes and machines. This occurrence could be attributed to their proximity to the jaw settings that define the field size, making them more susceptible to local fluence variability resulting from potential jaw positioning variations. Also, it could be attributed to the variations in beam steering over time.^31^

In both machines and imaging modes, FFF beams demonstrate limited areas with larger pixelwise standard deviations, closer to the center of the imager, compared to other regions of the EPID (Figure 4b). Flood field measurements of FFF beams were found to have greater variation in monitor units per image. The monitor units per acquisition were found to be (22.39 ± 0.07) MU for all flattened beams, representing a standard deviation of 0.3%, (55.71 ± 0.50) MU for 6FFF beams, corresponding to a standard deviation of 0.9%, and (95.89 ± 1.25) MU for 10FFF beams, representing a standard deviation of 1.3%. FFF beams have a conical profile with maximum intensity on the central axis and may experience variations in beam steering over time.^31^ The combination of conical beam shape, greater variation in exposure per image, and possible long‐term beam steering variation might have resulted in areas with greater pixelwise standard deviations, closer to the center of the imager, for FFF beams. No other discernible patterns are observed in the distribution of pixelwise flood field standard deviations.

Integrated over the entire EPID plane flood field signals show high correlation between energies in dosimetry mode, with r≥0.94 and r≥0.92 in Units A and J, respectively. The correlation coefficients between energies are significantly lower in continuous mode, with r≥0.57 in Unit A and r≥0.66 in Unit J. Best linear fits of integrated across the EPID plane flood field measurements demonstrated small increase over 2 years, in the range from 0.1% to 0.5% depending on the imaging mode and beam energy. At the same time, the determination coefficients have small values, demonstrating weak linear regression and practical absence of a trend. There was no correlation found between the flood fields measured in different imaging modes, in the same linac, for the same energy.

The EPID operated in dosimetry mode exhibits robust short‐term reproducibility, with a standard deviation ranging between 0.04% and 0.06% of the measured signal, which is comparable to the ion chamber (0.03%–0.05%) and MatriXX (0.02%–0.06%) values. Conversely, when the EPID operates in continuous acquisition mode, short‐term standard deviations increase by an order of magnitude, ranging from 0.32% to 0.52%. These values are comparable to the long‐term standard deviation observed in flood field measurements over the entire two‐year period.

Long‐term variation of the EPID response can be reduced by the linac output correction using the secondary absolute detector measurements. The standard deviation of the linac response showed reduction from 0.44%–0.51% to 0.28%–0.34% depending on the machine, beam energy, and the refence detector. The reduced long‐term values of standard deviation are greater than the short‐term standard deviation derived from the measurements taken on the same day. The decreased standard deviation range of 0.28%–0.34% represents the EPID response variation isolated from the fluctuations in linac output. These values align with findings from various publications by the other research groups.^19^, ^20^, ^21^, ^22^, ^23^, ^24^, ^25^, ^26^, ^27^ In particular, they show correspondence to the results of Barnes and Greer, who reported a‐Si 1200 EPID readings demonstrating up to a 0.6% drift when compared to reference absolute detectors.^25^ The observed long‐term variation of the EPID response corresponds to the clinical TG‐142 requirements, as the EPID response variation isolated from the fluctuations in linac output does not exceed 2%. Based on the observed long‐term variation in EPID response, there is no reason to revise the imager calibration frequency.

The comparison of the pixel sensitivity matrices acquired over a 24‐month interval shows 97.8% and 99.3% of pixels with differences below 1%, for Units A and J, respectively, and 100% of pixels with differences below 2% for both machines. There is a small fraction of pixels that demonstrates greater changes (over 1%) in sensitivity over time, compared to the rest of the imager. Their presence can be related to the construction of the phosphor layer of the EPID^23^ and the long‐term stability of the electronic readout circuit.^19^

The pixels that show greatest change can be viewed in Figure 10a,b as a combination of speckle and linear patterns. These patterns have different origins.^18^, ^19^, ^20^, ^21^, ^22^, ^23^ The detective phosphor layer of every a‐Si EPID has natural imperfections due to the manufacturing process.^18^ Their presence results in a small fraction (usually no more than 2%–3%) of pixels, randomly scattered across the detector plane, that may demonstrate both short‐term and long‐term instability of the measured signal.^18^, ^23^, ^30^ These pixels result in speckle patterns observed in signal variation images, and it has been previously established in the literature that the structure of the phosphor layer of the EPID causes these patterns.^23^

Also, a‐Si EPIDs are designed to have several electronic segments connected to their individual separate amplifiers.^19^ Stability of electronic amplifiers and multiplexers may degrade over time, resulting in step‐wise changes in sensitivity at the borders between image segments, and a strong visual enhancement of these borders.^18^, ^19^ The observed linear patterns may result from these long‐term changes to the EPID electronics. It was not possible to compare location of the observed linear patterns with the borders of the electronic EPID segments because the details of the proprietary a‐Si 1200 EPID design were not known.

The method used to determine PSM has a short‐term repeatability of 95% of pixels within 0.21% with the median less than 0.1%, according to the authors.^30^ While there exists a fraction of pixels that indicate long‐term changes surpassing these values, overall, the PSM demonstrates high stability throughout the 2‐year period.

## 5 CONCLUSION

The long‐term dosimetric performance of the a‐Si 1200 EPID detector was evaluated, focusing on output and reference field measurements. Both dark and flood calibration fields demonstrate good constancy over a 2‐year time period. Dark field measurements show smaller deviations than flood field measurements. The dark field shows greater signal variation in dosimetry mode, while the flood field shows greater variation in continuous mode. The pixel sensitivity matrix of the EPID detector showed constancy over time. The EPID response compared to a water‐equivalent detector measurement also remained stable over the 2‐year period (i.e., no significant drift). The results of the evaluation correspond to similar studies found in literature and confirm high detector stability and high reproducibility of the investigated model of EPID detector over an extended period of time. The long‐term variation of the EPID response was within clinical tolerance, and the detector was shown to be stable and reproducible for routine clinical dosimetry.

## AUTHOR CONTRIBUTIONS

Ivan Kutuzov: Participated in design of the experiment. Collected the data—measurements of EPID images: dark field, flood field, output measurements, and PSM. Collected reference linac output measurements using absolute dosimeters: ion chamber and ion chamber array. Wrote the Matlab code to perform analysis. Performed the analysis. Wrote and reviewed the manuscript. Dr Ryan Rivest: Designed the experiment. Reviewed the measurements. Reviewed the analysis tools and interpreted the results. Reviewed the manuscript. Dr Eric VanUytven: Designed the experiment. Reviewed the measurements. Reviewed the analysis tools and interpreted the results. Reviewed the manuscript. Dr Boyd M.C. McCurdy: Conceived and designed the idea of experiment. Provided overall guidance and leadership of the experiment through discussions with other authors. Reviewed the results of experiment. Reviewed the manuscript

## CONFLICT OF INTEREST STATEMENT

The authors declare no conflicts of interest.
